# Exploring athletes’ experiences and perspectives on an educational program for athletes in the Pacific Islands

**DOI:** 10.3389/fspor.2024.1388689

**Published:** 2025-01-07

**Authors:** Hee Jung Hong, Sungkyung Kim, Brian Minikin

**Affiliations:** Faculty of Health Sciences and Sport, University of Stirling, Stirling, United Kingdom

**Keywords:** athlete personal development, educational program for athletes, sport in the Pacific Islands, sport education, regional sport initiatives

## Abstract

This exploratory study aims to understand athletes’ experiences and perceptions of the “Voices of Athletes” (VOA), an athlete support program specifically designed and implemented in the Pacific Islands. Utilizing a mixed-methods approach, combining questionnaires and short interviews, data were gathered from 414 athletes (questionnaires) and 104 athletes (interviews) during the 2019 Pacific Games in Samoa. While a Chi-square test was used for quantitative analysis to assess the association between familiarity with the VOA and interest in learning more about the program, content and thematic analyses were employed to qualitatively examine participants’ perceptions and attitudes towards the VOA. The Chi-square result indicated that although the majority of athletes were initially unfamiliar with the VOA, there was a significant association between familiarity and interest in learning more about the VOA. Accompanied by the results from the quantitative data analysis, the results of the content and thematic analysis demonstrate that athletes who participated in the VOA onsite reported positive experiences and acknowledged the program's benefits in terms of personal growth and development. Participants emphasized the educational and informative aspects of the VOA, highlighting its role in increasing athletes’ awareness of contemporary social issues, empowering them to make informed decisions, and nurturing leadership skills within their communities. The results also highlighted the potential for integrating VOA into school curricula and using it as a model for support initiatives in other countries. Expanding the program could promote personal development, responsible decision-making, and leadership among a wider young population. Overall, the VOA offers significant benefits and has strong potential for broader application and customization across diverse environments. This can significantly contribute to the comprehensive growth of athletes and young people worldwide.

## 1 Introduction

Athlete support programs designed to assist athletes with career development and transitions beyond their athletic careers have been established and implemented across the world (1). Studies have shown that athletes face different challenges and obstacles during and after their athletic careers (2, 3), prompting scholars to advocate for sport governing bodies and sport organizations, such as National Olympic Committees (NOCs), to take responsibility for supporting athletes in managing both athletic and non-athletic careers to ensure their well-being [e.g., (1, 4, 5)]. As a result, sport governing bodies have developed career assistance programs (CAPs) with different formats to assist athletes in managing their athletic careers and preparing for life after sports (6). Despite the increasing attention given to organizational support in high-performance sports, research in this area remains limited compared to social support for high-performance athletes [e.g., (7, 8)]. The existing body of literature tends to focus on specific elements of organizational support, which may not provide a comprehensive understanding of the overall support structure for athletes. For instance, Maier et al. (9) identified three critical areas of organizational support within high-performance sports settings: family integration, second career support, and private problem support. While these areas are crucial, they do not encompass the full range of support services that athletes may require. Studies have examined organizational support with a focus on other aspects (10), such as injury prevention and provision of socioeconomic services (11), and athletes’ involvement in non-athletic careers/activities (12). However, a more in-depth analysis of the literature shows that the effectiveness of organizational support programs often depends on the unique needs and circumstances of individual athletes, as well as the cultural and contextual factors that shape their experiences. As such, a one-size-fits-all approach to organizational support may not be sufficient to address the unique needs of high-performance athletes.

Research on athletes’ career development and the transition has been predominantly conducted in Australia, Europe, and North America (13). Park et al. (2) also noted that the majority of studies on athlete support systems were carried out in Western countries, with 10 in Australia, 45 in Europe, and 60 in North America. To address this geographical disparity, Hong and Coffee (1) examined sport career transition support programs in 19 different countries spanning Africa, Asia, Europe, North America, South America, and Oceania. Although their study offered a comprehensive overview of organizational support through CAPs across various continents, the countries investigated were primarily developed nations, such as Australia and New Zealand within Oceania. As a result, there is a significant gap in research exploring athlete support and CAPs in developing countries, particularly those in the Pacific Islands, excluding Australia and New Zealand. This lack of representation hinders the development of effective support strategies tailored to the unique needs and circumstances of athletes in these regions. Expanding research efforts to include developing countries in the Pacific Islands would offer a more comprehensive understanding of athlete support systems and services worldwide. It would also highlight how cultural, social, and economic factors could influence the success of structured support programs. In addition, by exploring the experiences and perspectives of athletes from underrepresented regions, the present study aims to foster more inclusive insights into career development and transition support for athletes. This effort seeks to improve the wellbeing and long-term success of athletes.

To the best of the authors’ knowledge, no CAPs have been identified in the Pacific Island regions, with the exception of Australia and New Zealand, comparable to those established in other countries for supporting athletes’ career development and transitions. However, by 2006, the Oceania National Olympic Committees (ONOC) had established its own regional sports education programs, known as the Oceania Sport Education Program (OSEP). Initially focused on educating coaches and sports administrators, OSEP also supported the emergence of new initiatives such as Voices of Athletes (VOA). The VOA was originally conceived by the managers of two athlete-cantered education programs introduced to Oceania: a drug education program developed by the Oceania Regional Anti-Doping Organisation and the Stop HIV initiative, jointly funded by the United Nations Programme on HIV/AIDS (UNAIDS), the South Pacific Commission, and ONOC. Established in 2004 as a pilot program by the World Anti-Doping Association (WADA) and ONOC, these programs joined forces in 2007 to create a platform for delivering messages directly to athletes.

The initial effort to engage athletes was launched in 2007 at the Pacific Games held in Samoa, where an education booth was established in the Games Village (14). This endeavor led to various athlete outreach activities at numerous national and regional sporting events, as well as in specific countries. These efforts proved successful in raising awareness and providing athletes with opportunities to discuss various issues affecting their lives. While the VOA represents a step forward in athlete support within the Pacific Islands, further research and expansion of such programs are needed to address the unique challenges and needs of athletes in this region. By exploring athletes’ experiences and perceptions on the VOA and similar initiatives, the present study can contribute to the development of comprehensive and culturally sensitive support strategies that foster the holistic development and wellbeing of athletes in the Pacific Islands and beyond.

This paper, part of a project focused on the VOA, aims to explore the awareness of athletes in the Pacific Islands regarding the VOA, as well as to examine their experiences and perceptions of the program's activities. The research questions are: (a) how familiar are athletes in the Pacific Islands with the VOA? (b) what are the experiences and perspective of the athletes in relation to their participation in the VOA, and which elements of the VOA should be highlighted or improved? (c) what factors will contribute to the further development and sustainability of the VOA? By answering the research questions, this study intends to offer insights into an athlete support program in the Pacific Island region, an area that has not been thoroughly explored in comparison to the CAPs in other countries documented in previous studies. Understanding the impact of the VOA on athletes in this region will not only help inform the ongoing development and implementation of the program but also contribute to the broader knowledge of athlete support systems in diverse contexts.

### 1.1 Athlete support programs

Researchers focusing on athletes’ career development and transitions have recognized the necessity for athletes to receive support in preparing for their transitions during and after their athletic careers, as well as in coping with challenges and obstacles they may encounter (1, 13, 15, 16). In a study with Australian athletes, Albion and Fogarty (17) discovered that those who engaged in the Athlete Career and Education (ACE) program for an extended period were more motivated to participate in career decision-making. While the athletes in Albion and Fogarty's (2003) study demonstrated high levels of awareness regarding the various aspects of the ACE program, the specific areas in which they participated and the degree of involvement remained unclear (15). National governing bodies (NGBs) have been deemed responsible for supporting athletes in career development and transitions (5), as the development of athletes’ life skills may be delayed due to their intensive commitments to training and competing (16). Anderson and Morris (4) argued that sporting organizations should encourage athletes to develop life skills and a well-rounded identity, allowing them to lead a balanced life. They further suggested that these organizations could play a role in creating an appropriate environment for athletes to manage their career development and transitions by developing athlete lifestyle programs.

Career Assistance (CA) aims to guide athletes in handling various obstacles tied to their sports and non-sports careers (18). As an athlete support intervention, CA allows athletes to balance their athletic pursuits alongside other life commitments (19). Interventions can be categorized into two distinct approaches: proactive/supportive and crisis/poor coping (18). From a proactive/supportive angle, interventions assist athletes in foreseeing and readying themselves for upcoming challenges related to transitions and managing dual careers. In contrast, from the crisis/poor coping perspective, interventions help athletes assess crisis incidents or difficult situations they have experienced, equipping them to explore and adopt successful coping tactics (19). Such interventions have contributed to the formation of career assistance programs (CAPs), with the first CAP introduced in the late 1980s to help retired athletes transition out of sport [e.g., the Olympic Job Opportunities Program (3);]. Since then, sports organizations around the world have launched CAPs to facilitate athletes’ career growth and transitions (6). However, it is important to recognize that such CAPs are not solely focused on assisting athletes in career development and transition, but also encompass personal growth and educational support (1).

In recent years, research on CAPs has advanced significantly, with a growing body of literature examining various aspects of these programs. For instance, Hong and Coffee (1) carried out a study examining CAPs in 19 countries, illustrating the extensive development and implementation of CAPs worldwide since Lavallee et al. (20) initially reviewed CAPs in seven individual countries. Their analysis of CAPs centered on five main aspects: (a) identifying the organization accountable for providing sports career transition intervention programs at the government level; (b) summarizing the overarching strategies of sports career transition intervention programs; (c) describing the activities and content of these intervention programs; (d) identifying the practitioners responsible for delivering the intervention programs; and (e) examining whether sports organizations offer training and development programs for practitioners who cater to high-performance athletes. Based on their results, Hong and Coffee (1) emphasized the importance of additional research into international comparisons of CAPs and the available training programs for CAP practitioners. This highlights the continuous need for a more in-depth understanding of CAP implementation and efficacy in assisting athletes during their career transitions.

However, their study did not investigate this subject from the perspective of athletes as users. This highlights the need for further research to explore the experiences and perspectives of athletes to better understand the impact of such programs in various contexts. In a more recent study, Torregrossa et al. (19) conducted a comprehensive analysis of CAPs, exploring elements such as definitions, goals, delivery approaches, and the stages of emergence, growth, and implementation. The researchers proposed a classification system for CAPs, including: (a) Holistic CAPs for elite athletes, (b) Sport-specific CAPs for professional athletes, and (c) Dual CAPs for student-athletes. This categorization was established based on an examination of various CAPs (*n* = 8) from multiple countries and organizations, such as Australia, Belgium, Canada, the UK, and the US. This classification framework proves useful for comprehending CAPs not evaluated in their study, especially those implemented outside of Europe and North America. Although recent studies have investigated CAPs [e.g. (1, 19)], research in this field remains scarce, as highlighted by Stambulova and Ryba (18). Thus, there is an urgent need for more research aimed at evaluating CAPs and applying the insights gained to practical settings (19).

Expanding on the existing literature, there are several aspects of CAPs that need further investigation. For instance, the influence of cultural and contextual factors on the development and execution of athlete support programs has not been thoroughly studied. Exploring how culture and context shape the experiences and perceptions of athletes and practitioners involved in CAPs can offer valuable insights. This knowledge can help customize programs to meet the unique needs of different groups more effectively (21). In this regard, Hong and Coffee (1) have expressed concerns that CAPs are still limited in certain regions, particularly in areas that might benefit the most from such support. As mentioned previously, no CAPs have been identified in the Pacific Island regions, with the exception of Australia and New Zealand, that are similar to those implemented in other countries to support athletes’ development. However, the Oceania National Olympic Committees (ONOC) has proactively initiated regional sports education programs tailored to the unique needs of the Pacific Island region. One of these initiatives is the Voices of Athletes (VOA), which seeks to address the gap in athlete support services in this region. In the following section, we will explore the VOA, focusing on its objectives, how it is implemented, and its potential influence on the personal and professional growth of athletes in the Pacific Island regions.

### 1.2 Voice of athletes (VOA)

Following the 2000 Sydney Olympic Games, the ONOC initiated a four-year plan aimed at enabling Pacific Island communities to create and implement their own sports initiatives (22). This approach resulted from a successful partnership between the Australian Government and ONOC, with the goal of creating sustainable education programs. These programs are to be carried out by local Sport Development Officers in every Pacific Island country (22). The plan recognized the importance of building the ability to effectively use resources from Olympic Solidarity, national governments, and international sports governing bodies (23–25). This awareness emerged from workshops and studies showing that, despite significant funding for sports, tangible progress in the Pacific was limited (26–28). As a result, the emphasis shifted towards nurturing Pacific individuals through sport, rather than exclusively for sport (27). As ONOC's development plan progressed (29), the comprehensive development of athletes became a central focus. This led to the establishment of Voices of the Athletes (VOA), which transitioned into an advocacy role after being incorporated into ONOC's Athletes Commission in 2009 (30).

The VOA is designed to motivate athletes to be positive role models, summarized by the slogan “*Be a Leader*.” The program includes four unique initiatives:

*Play True*: This component evolved from the original anti-doping education programs promoted by WADA and has been adapted to suit the context of the developing sports systems in the Pacific Region. It aims to instill a culture of clean sport among athletes.

*Stay Healthy*: Originating from the initial *Stop HIV* initiatives, this aspect of the program now encompasses broader health and lifestyle issues, such as non-communicable diseases and overall well-being, to encourage athletes to adopt healthier lifestyles.

*Go Green*: Adopted from global environmental initiatives established by the IOC, this element promotes sport as a vehicle for spreading messages related to conservation and minimizing the impact of human activity on the planet, urging athletes to be environmentally responsible.

*Play Safe*: Stemming from athletes’ concerns about harassment and abuse, this initiative focuses on safeguarding athletes, particularly those vulnerable to abuse from authority figures. Play Safe responds to the growing attention worldwide on the need to protect athletes and create safe sporting environments.

The VOA was among the first programs of its kind globally, emerging directly from the need for coordination and cooperation among multiple agencies operating in resource-limited settings (30). Most importantly, it serves as an educational platform to support athletes’ personal development, empowering them to become well-rounded individuals who can positively impact their communities and the world of sport. By continually adapting and expanding its initiatives, the VOA demonstrates the potential for similar programs to address the diverse needs of athletes in various regions and foster holistic development across the athletic community.

As the VOA continues to evolve and address the multifaceted needs of athletes in the Pacific Islands, it exemplifies the potential for similar programs to foster holistic development and positive change throughout the global athletic community. By embracing adaptability and focusing on the personal development of athletes, the VOA has become a critical support system for athletes in this region, inspiring them to become agents of change within their communities and beyond. In light of these successes, the current study aims to examine the VOA, which has been in operation since 2007, serving active athletes from various Pacific Island nations and across a range of sports. By exploring the perspectives of athletes in this diverse context, the study seeks to offer invaluable insights into the adaptability and impact of such programs across different populations, sports, and regions. It is hoped that this research can contribute to an enhanced understanding of the factors that influence the efficacy of athlete support programs and how they can be optimized to better facilitate the personal and professional development of athletes in a variety of settings.

### 1.3 Theoretical underpinnings

Bronfenbrenner's (31) ecological systems theory consists of four environmental levels—microsystem, mesosystem, exosystem, and macrosystem—each impacting an individual's development differently. The microsystem, or Level 1, includes the immediate environment where a child or adolescent closely interacts, such as classrooms, playgrounds, homes, and neighborhoods. Bronfenbrenner (31) defined the microsystem as “a pattern of activities, roles, and interpersonal relations experienced by the developing person in a given setting” (p. 22). The mesosystem, Level 2, involves the interactions between two or more settings in which the individual actively participates. According to Bronfenbrenner (31), this includes relationships between home, school, and neighborhood peer groups. The exosystem, or Level 3, represents settings that do not involve the person as an active participant but affect or are influenced by the individual's immediate environment. For example, a person's home experiences might be influenced by their spouse's work experiences. Lastly, the macrosystem, or Level 4, pertains to the consistencies in lower-order systems (micro-, meso-, and exo-) that exist at the subculture or cultural level. In other words, the macrosystem involves the broader cultural context surrounding the individual, including societal belief systems, cultural norms, ideologies, policies, or laws that indirectly influence the person. Macrosystems evolve over time, reflecting their temporal development (31).

Bronfenbrenner's ecological systems theory provides a valuable framework for examining the intricate interactions between athletes, athlete support programs, and the broader sociocultural context in the Pacific Island region (31). Applying this ecological model to the current study enables the exploration of how various environmental systems influence the VOA's impact and shape athletes’ personal and professional development within this unique setting. Within the context of Bronfenbrenner's ecological systems theory, researchers can investigate different aspects of athletes’ development. At the microsystem level, researchers can explore immediate relationships between athletes, families, coaches, and support staff (32). Analyzing interactions within these microsystems allows for the identification of factors contributing to successful athlete engagement with support programs such as the VOA or other CAPs, including the quality of coach-athlete relationships (33) and the support offered by family members (34, 35). The mesosystem level enables the investigation of how different microsystems interact, such as the connections between athletes’ families and sports organizations, and how these interactions influence the implementation and effectiveness of athlete support programs (36). At the exosystem level, researchers can examine the roles of sports organizations, funding agencies, and national governing bodies in developing, implementing, and supporting athlete programs in the Pacific Island region, as well as the barriers and facilitators that emerge from these relationships (1, 18). Applying the macrosystem level allows for the examination of cultural and social norms shaping athlete development and support initiatives in the Pacific Island region. By considering how these broader contexts influence the design and implementation of athlete support programs, valuable insights can be gained for tailoring programs to better meet the unique needs of local athletes (21).

The present study is particularly suitable for exploring the exosystem and macrosystem levels, as these levels provide valuable insights into the broader environmental factors influencing athletes’ experiences and perceptions of the VOA. By focusing on the exosystem level, this study can investigate how sports organizations/governing bodies contribute to the development and implementation of athlete support programs in the Pacific Island region. This perspective can show the complex interplay between the stakeholders and highlight potential areas for improvement, collaboration, or alignment of goals and strategies to enhance the effectiveness of the VOA and other support initiatives. Furthermore, by examining the macrosystem level, the study can explore the cultural and societal influences that shape athletes’ experiences, as well as the design and implementation of athlete support programs in the Pacific Island region. This focus on the macrosystem level is crucial for understanding the unique cultural and social contexts in which the athletes and support programs operate, allowing for a more nuanced and tailored approach to program design and implementation. By acknowledging and addressing these broader factors, the study can contribute valuable knowledge to guide the development of athlete support programs that are better suited to the specific needs, values, and traditions of athletes in the Pacific Island region, eventually enhancing the support and resources available to these athletes throughout their personal and professional development. Examining athlete support programs such as the VOA at both the exosystem and macrosystem levels can theoretically contribute to literature by providing a more comprehensive understanding of the interplay between athletes, their support systems, and the broader environmental context. Practically, these insights can help sport organizations, governing bodies and professionals develop more effective, targeted, and culturally sensitive support initiatives that cater to the unique needs of athletes across diverse contexts, enhancing overall athlete wellbeing and success in their sporting careers.

## 2 Materials and methods

This study employed a two-phase approach for participant engagement at the 2019 Pacific Games: (a) questionnaire collection and (b) conducting short interviews. In the first phase, a mixed-methods approach was utilized, incorporating a questionnaire featuring both closed and open-ended questions. One advantage of using self-administered questionnaires with open-ended questions is the potential reduction in bias compared to face-to-face interview inquiries (37). The questionnaire items were developed through a series of discussions among the research team and officers from the ONOC. Given that data collection took place during the 2019 Pacific Games, the research team intentionally designed straightforward questions to avoid overwhelming the athletes or creating undue pressure to complete the questionnaire within a limited timeframe. In the second phase, to explore athletes’ experiences with the VOA, interviews were conducted at the exit of the VOA booth during mealtimes. Although these two phases were differentiated, data collection was carried out concurrently by the first author, depending on the number of athletes present at meal tables and the VOA booth.

### 2.1 Participants

Pacific Games aim to “promote a unique, friendly world-class competition and Games and to develop sport for the benefit of the people, the nations and the territories of the Pacific Community” (38, p.3). Island nations and territories that are part of the Pacific Community qualify for membership in the Pacific Games Council. The member countries include American Samoa, Australia, Cook Islands, Federated States of Micronesia, Fiji, Guam, Kiribati, Marshall Islands, Nauru, New Zealand, Palau, Papua New Guinea, Samoa, Solomon Islands, Tokelau, Tonga, Tuvalu, and Vanuatu. Associate members include New Caledonia, Niue, Norfolk Island, Northern Mariana Islands, Tahiti, and Wallis & Futuna (38, 39).

The first author collected the completed questionnaires immediately at the athletes’ meal tables. Participants came from 16 of the 24 countries represented at the Games. Eight countries that were not represented in the sample include Federated States of Micronesia, Kiribati, Marshall Islands, Nauru, Niue, Northern Mariana Islands, Tuvalu, and Wallis & Futuna. In addition, athletes from the Netherlands, Spain, and the USA were excluded on the grounds that they were representatives of specific Pacific nations but not residing in Pacific Island countries. While they competed on behalf of Pacific nation teams, they acknowledged the origin of their birthplace and confirmed that they planned to their own nations after the Games. While these were unforeseen circumstances, the research team decided to focus on athletes based in the Pacific Nations. In total, 414 athletes participated in the survey, representing over 10% of the approximately 3,500 athletes who competed at the Pacific Games.

To investigate athletes’ experiences with the VOA, short interviews were conducted at the exit of the VOA booth. The participant selection criteria included having participated in the VOA and being proficient in English. A total of 123 athletes and support staff members were interviewed, as detailed in Table 2.

The athletes represented 16 different countries, including American Samoa, Australia, Cook Islands, Fiji, Guam, New Caledonia, New Zealand, Norfolk Island, Palau, Papua New Guinea, Samoa, Solomon Islands, Tahiti, Tokelau, Tonga, and Vanuatu, with three participants having missing information. Among these countries, the official language in both New Caledonia and Tahiti is French. Thus, only athletes from these regions who could speak English were able to participate. They competed in 19 different sports, such as archery, badminton, basketball, boxing, canoeing, cricket, football, golf, judo, netball, rugby, sailing, squash, swimming, taekwondo, tennis, touch rugby, volleyball, and weightlifting, with five participants having missing information.

### 2.2 Procedure

Following institutional ethical approval (GUEP659), data collection for this study was carried out during the Pacific Games 2019 in Samoa between 9th and 15th July 2019. To ensure clarity and convenience for participants in the questionnaire due to the limit time, group consent was first obtained from the head coaches before distributing the questionnaire to each team. In cases where teams included athletes under 18, the lead author further ensured consent by confirming with both the head coaches and the athletes directly. The head coaches informed that the research team did not require their names to maintain anonymity. Subsequently, the lead author explained the project details to each team prior to sharing the questionnaires. Athletes who chose to participate completed the questionnaire. While it is common practice for each participant to provide individual consent, this approach was reviewed and approved by the university's ethics committee, given the specific context. The questionnaire was completely anonymous, including no identifying information, and participants were explicitly informed that their participation was voluntary, ensuring that ethical standards were adhered to. For the short interviews, verbal consent was recorded before each interview to ensure participants’ convenience and streamline the process given their limited time. The Voices of Athletes (VOA) booth was strategically positioned in an area where athletes had their meals, allowing the first author to collect data during lunch or dinner time. The questionnaire included three main questions, along with demographic information (see Table 1): (a) Are you familiar with Voice of the Athletes? (b) Have you participated in Voice of the Athletes before? (c) Are you interested in learning about Voice of the Athletes? Based on their responses to the second question, athletes were asked to provide either information about the positive aspects and areas for improvement of the VOA program or reasons for not participating in the VOA. Those who answered the third question were asked to provide reasons for their lack of interest if they selected a “not decided” option.

**Table 1:** Demographics.

Variable	*n*	%
Gender		
Female	214	52
Male	196	48
Prefer not to say	2	1
Age group		
Under 18	73	18
18–25	194	47
25–30	92	22
Over 30	51	12
Education		
High school	122	30
College	108	27
Undergraduate degree	97	24
Post-graduate degree	32	8
Other	46	11
Nationality		
Guam	5	1
French Polynesia/Tahiti	21	8
Spain	1	0.2
Samoa	50	12
New Zealand	12	3
Vanuatu	43	10
Papua New Guinea	38	9
Tonga	23	6
New Caledonia	25	6
Nauru	15	4
Northern Mariana Islands	7	2
Cook Islands	16	4
USA	2	1
Palau	9	2
Tokelau	10	2
Solomon Islands	24	6
Fiji	53	13
American Samoa	26	6
Australia	13	3
Netherlands	1	0.2
Unknown	1	0.2

Missing data = Gender (*n* = 2), Age (*n* = 4), Education (*n* = 9), Nationality (*n* = 2).

**Table 2:** VOA participants onsite.

Roles	Numbers and %	Nationalities	Gender	
			Male	Female
Athletes	104 (84.6%)	16	42	62
Support staff members	19 (15.4%)	9	10	9
Total number	123 (100%)			

**Figure 1:**
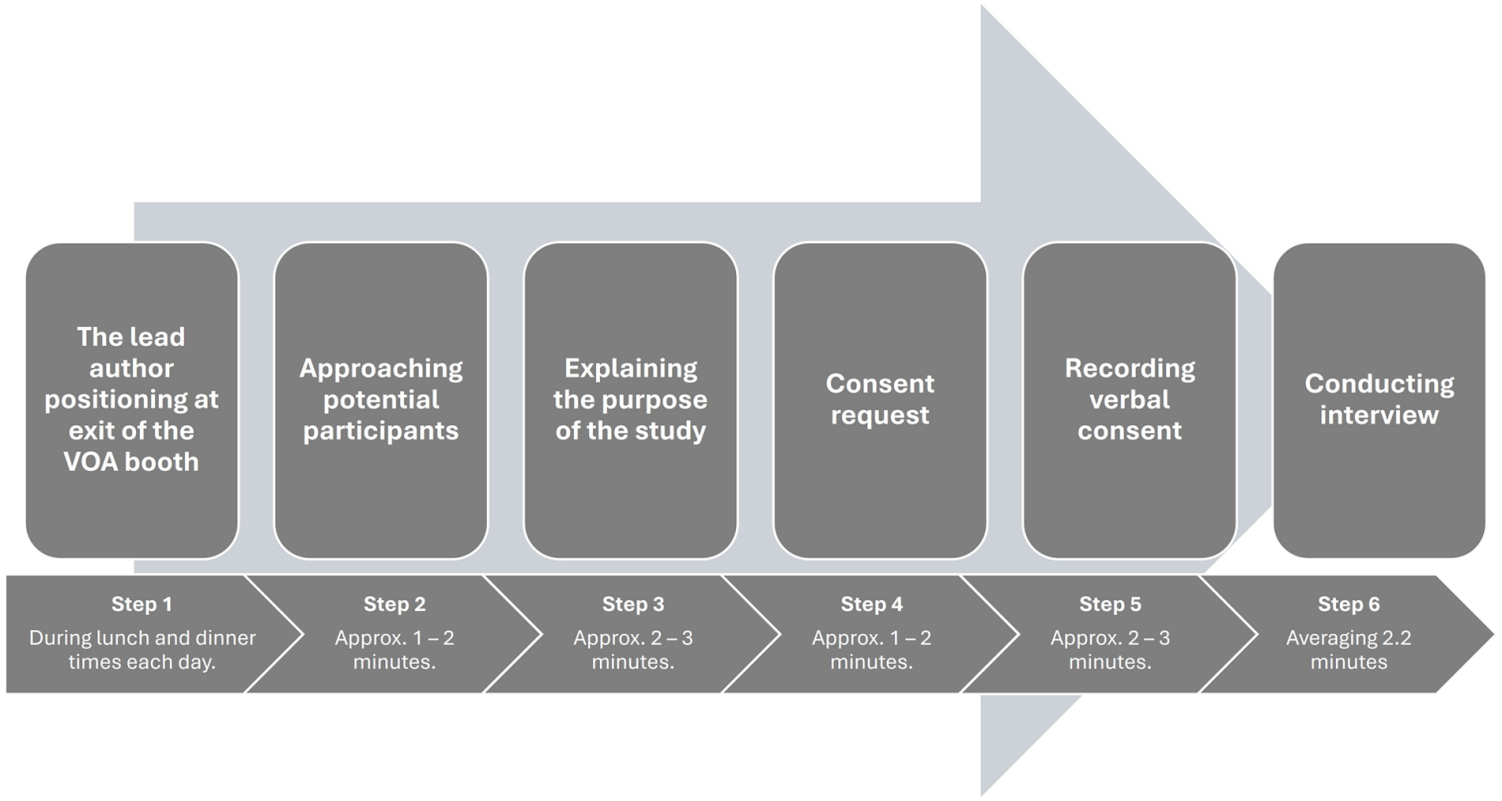
The procedure for interviews.

Short interviews were utilized to allow research participants to share their first-hand experiences of the VOA, rather than relying on observations of a given situation (40). The procedure for the interviews is presented in Figure 1.

This approach enables researchers to obtain each participant's personal account. The interview guide was designed to address our research questions, exploring participants’ experiences while minimizing the time required for interviews, and effectively maximizing the quality of their responses within this limited timeframe. The interviews focused on the following topics: (a) sport background (what sport do you compete in, and how long have you been involved in it?), (b) motivation to participate in the VOA (what motivated or inspired you to participate in the Voices of the Athletes?), (c) overall experience of the VOA (how would you describe your overall experience with the Voices of the Athletes?), (d) positive aspects of the VOA (what aspects of the Voices of the Athletes program did you find most valuable or beneficial?), (e) lessons learned from the VOA (what are some key lessons or insights you gained from participating in the Voices of the Athletes?), and (f) areas for improvement (in your opinion, what could be improved or added to the Voices of the Athletes program to enhance its effectiveness?). This interview guide was consistently applied to ensure uniformity throughout the interviews (41). Given that participants were interviewed at the exit of the VOA booth and had limited time for meals and VOA participation between their training and competitions, each interview was relatively brief, averaging 2.2 min in duration. Short interviews, often referred to as “brief” or “micro-interviews,” can be effective in time-sensitive situations, such as sports mega events. These interviews offer efficiency, allowing researchers to collect data without imposing major time constraints on busy athletes and staff. Short interviews also minimize participant fatigue and maintain engagement, leading to more accurate and higher-quality data. In high-stress environments such as sports mega events, these interviews ensure reliability and usefulness of the data. Furthermore, they capture participants’ immediate reactions and experiences, offering valuable insights into the dynamic nature of their experiences during such events (42–44).

### 2.3 Data analyses

The quantitative data from the responses was analyzed using SPSS Statistics 29.0 version. For the dichotomous items (i.e., Yes or No responses), the current study utilized Pearson's Chi-squared (*χ*^2^) test to examine the association between categorical variables. Specifically, we assessed the relationship between familiarity with the VOA and interest in learning more about the program. We performed additional analyses, including the Chi-square continuity correction (*χ*^2^) and the likelihood ratio (G^2^), to confirm the robustness of the results. Fisher's exact test was also conducted to verify these associations, providing an added level of reliability in case of small, expected frequencies in the data. For open-ended questions related to positive aspects, areas for improvement, and reasons for not being interested in participating in the VOA, a deductive approach (45) was employed based on the research questions. Deductive analysis enables researchers to identify key concepts informed by existing literature within the collected data (46, 47), using a framework, such as research questions, to guide the study (48). Adopting a deductive approach, initial codes were developed from literature relevant to the study topic or from “what is known about the phenomenon of inquiry, aided by the research aims, research questions, and interview questions” (45, p. 391).

The audio-recorded interview data was transcribed verbatim to facilitate accurate data analysis. While interviews were conducted at the event venue, we took measures to capture responses as clearly as possible, despite the occasional background noise. Since we recognized the significance of audio clarity, we selected the quietest available areas at the venue and minimized interruptions as best as possible given the circumstances. As a result, all interview audio files were captured clearly. In terms of the transcription process, we used a professional transcription service to ensure precision in capturing participants’ words. Each transcript was then carefully reviewed by research team members to confirm accuracy, providing an additional layer of validation. A total of 141 pages of transcripts were generated from interviews conducted at the exit of the VOA booth (*n* = 123). Data analysis transitioned from an inductive to a deductive approach (49). Inductive analysis allowed new themes to emerge from the data, while the deductive analysis incorporated pre-existing categories or frameworks, such as those based on the research questions, to contextualize relevant quotes within the identified themes (50). Thematic analysis was employed to scrutinize the transcribed data, enabling researchers to discern patterns within the qualitative interview data (51). The six-phase process proposed by Braun and Clarke (52) guided the authors in their analysis. To ensure the rigor of the thematic analysis, a systematic and thorough checklist was followed (51). The authors initiated the data analysis and subsequently reviewed each other's work. Although they largely agreed on the identified themes and process, points of disagreement were resolved through a series of discussions until consensus was achieved. This “team consensus” promoted the trustworthiness of the data analysis process (53). Finally, deductive analysis was conducted to verify the validity of the analysis. This involved reviewing all transcripts and relevant quotes to confirm that the emerged themes accurately represented the data and participants’ perspectives (49).

In the context of the present study, a deductive analysis is well-suited for exploring athletes’ experiences with the VOA, as it allows researchers to build upon existing knowledge and theoretical frameworks, such as Bronfenbrenner’s ecological systems theory, specifically focusing on the exosystem and macrosystem levels. By utilizing research questions derived from literature and the ecological systems theory, the deductive approach ensures that the analysis remains focused and relevant to the study’s objectives. Moreover, this method facilitates the identification of patterns and relationships within the data that align with, challenge, or expand upon the established understanding of athletes’ experiences in the context of athlete support programs like the VOA, while taking into account the broader sociocultural and organizational factors that influence these experiences.

## 3 Results

In this section, we present a comprehensive analysis of the questionnaire data, organized under two primary sub-sections: “Familiarity with and Interest in the VOA” and “Positive Aspects and Areas for Improvement.” These sub-sections effectively capture participants’ initial engagement with and evaluation of the VOA. Alongside the questionnaire findings, we also discuss the results derived from the short interview data, which led to the identification of key notable themes: “Engaging and Informative Educational Experiences,” “Fostering Awareness of Social Issues,” and “Recommendations for Improved Delivery.” These themes reflect participants perspectives on the educational value of the VOA, their heightened consciousness of social issues, and their valuable insights on how to enhance the program's overall impact and effectiveness.

### 3.1 Familiarity with and interests in the VOA

The association between familiarity with the VOA program and interest in learning more about the program was examined using Pearson Chi-square (*χ*^2^) test, suitable for analyzing categorical variables. In the analysis of the Chi-square test, this study excluded 41 respondents due to missing values, utilizing a final sample size of 373 for the analysis (see Table 3). The result of analysis showed a significant association between familiarity with the VOA and interest in further engagement (*χ*^2^ [2] = 82.5, *p* < .001; see Table 4). Consistent results were obtained with the continuity correction (*χ*^2^ [2] = 82.5, *p* < .001), likelihood ratio (G^2^ [2] = 69.4, *p* < .001), and Fisher's exact test (*p* < .001), confirming the robustness of these findings.

**Table 3:** Interest in learning more about the voice of athlete (VOA) program by familiarit**y.**

Questions		Interest in learning more about VOA			
		Not interested in learning more	Interested in learning more	Not decided	Total (*n*)
Familiarity with VOA	Not familiar with VOA	57 (89.1%)	239 (82.7%)		296 (79.4%)
	Familiar with VOA	7 (10.9%)	50 (17.3%)	20 (100%)	77 (20.6%)
	Total (*n*)	64 (100%)	289 (100%)	20 (100%)	373 (100%)

41 respondents were excluded from the final analysis due to missing values.

**Table 4:** The results of the Chi-square test.

	Value (*p*-value)	*df*
Pearson Chi-square (*χ*^2^)	82.5*	2
Chi-square (*χ*^2^) continuity correction	82.5*	2
Likelihood ratio	69.4*	2
Fisher's exact test	*p* < .001	
The total sample size (*n*)	373	

* *p* < .001.

Given the significance across tests, including the robustness check provided by Fisher's exact test, the results highlight the importance of familiarity with the program in predicting interest in further engagement. These outcomes highlight the potential impact of increasing awareness and understanding of the VOA program as a method to enhance engagement and participation rates in the program.

### 3.2 Positive aspects and areas for improvement

Of the participants familiar with the VOA, 23% provided insights into the programme's positive aspects and areas for improvement. With regard to the positive aspects, participants highlighted three key factors. First, they appreciated the learning opportunities offered by the VOA, which enabled them to expand their knowledge on a range of topics, including environmental protection, doping, abuse and harassment, athletes’ empowerment and awareness of social issues, leadership, safety and health, and inclusivity. Second, participants valued the opportunity to interact with fellow athletes from different countries, fostering social connections and camaraderie during lunch or dinner breaks. Finally, involvement in the VOA programme enhanced participants’ understanding of their rights as athletes, as well as their responsibility to advocate for athlete welfare and wellbeing.

Participants also offered valuable insights into potential areas for improvement within the VOA. Four primary areas were identified. First, language barriers emerged as a challenge, as the program was facilitated exclusively in English. Although English is an official language in many Pacific Island countries, some athletes primarily speak French or other regional languages. Second, participants enjoyed the various activities at each station and suggested that incorporating additional games or interactive elements would make the learning experience more engaging and enjoyable. Third, athletes mentioned that relocating the VOA booth to the Games Villages would have improved accessibility, as they were only able to visit the booth during lunch and dinner breaks, which provided limited opportunities. Finally, participants recommended expanding the programme to schools in each country, as they valued the educational components of the VOA.

### 3.3 Engaging and informative educational experiences

Upon being asked about their overall impression of the VOA after completing some or all of the stations, participants expressed that it served as a positive distraction from competition and offered a valuable opportunity to interact with athletes from other countries. Importantly, the majority of participants emphasized the educational and informative aspects of the VOA. It was noteworthy that participants were enthusiastic about learning new information and keen to share their newfound knowledge with others. Athlete 51 stated, “It's highly informative—it teaches you to stay away from alcohol and cigarettes to be healthy and active, and to avoid non-communicable diseases.”

Athletes highlighted the potential of the VOA as an educational tool for young people in the Pacific Islands. For instance, Athlete 30 remarked, “It's an effective way to inform the youth about important issues, particularly with regard to sexually transmitted diseases and such.” Athlete 10 suggested that the VOA should be made available online to enable school children to access the valuable information presented to the athletes. The engaging and enjoyable learning strategies employed by the programme also contributed to participants’ positive experiences. Athlete 85 shared, “It was really useful, beneficial for us, and educational. Plus, it was fun at the same time. I genuinely enjoyed it.”

### 3.4 Fostering awareness of social issues

Participants emphasised the valuable opportunities provided by the VOA to share, develop knowledge, and raise awareness of various social issues. The VOA featured four distinct stations: Go Green, Play Safe, Play True, and Stay Healthy. Among these, the “Stay Healthy” initiative received the most mentions from participants, as detailed in Table 5. This station focused on major public health issues in the Pacific Islands, such as non-communicable diseases (NCDs), human immunodeficiency virus (HIV), alcohol and smoking abuse, physical inactivity, and hygiene-related concerns. Participants engaged with interactive games, such as crossword puzzles, to develop their understanding of these issues and learn strategies for maintaining good health. Athletes acknowledged the significance of learning about NCDs and other health concerns, as they witnessed the negative impacts of these issues on their sports commitments and performance.

**Table 5:** The most impressive/interesting stations.

Stations	Numbers and %
Go Green	10 (8.1%)
Play True	26 (21.1%)
Play Safe	29 (23.6%)
Stay healthy	36 (29.3%)
Others	22 (17.9%)
Total	123 (100%)

The “Play Safe” station raised awareness of harassment and abuse in sports. Participants who found this station particularly impactful appreciated the opportunity to become informed about these issues. Athlete 57 emphasised the importance of voicing concerns about abuse and harassment in the Pacific, noting that coaches and players often remain silent on the matter. Participants suggested that education on these issues should be provided to young athletes early in their careers. Participants also valued the role of sport and the VOA in delivering crucial messages to young people in an engaging manner. The “Play True” station focused on doping education, which was the original emphasis of the VOA. Participants appreciated the use of virtual reality technology to help them understand the importance of anti-doping education and the doping test procedure. Athletes from the Pacific Islands found this experience particularly valuable, as many participants were unfamiliar with the doping testing procedure. The recently added “Go Green” station attracted participants who were interested in environmental conservation and adopting eco-friendly lifestyles. Participants praised the interactive nature of the activities, which effectively conveyed important messages while being enjoyable.

The remaining participants (*n* = 22) did not specify a preferred station but emphasised that the overall programme provided them with ideas and insights on sharing the messages they learned with young people in their countries, reflecting the VOA’s primary objective of promoting leadership among athletes.

### 3.5 Suggestions for enhanced delivery and accessibility

Although participants had a positive and beneficial experience with the VOA, they also offered valuable insights into potential areas for improvement. They enjoyed learning useful information through games at each station and recommended adding more activities and stations to accommodate more athletes within limited timeframes and to provide additional enjoyable distractions from competition and training. Participants also suggested that a larger, more spacious venue would be helpful, as the entire VOA booth was set up at the back of the meal area.

Another recommendation was to locate the VOA in various locations, such as the athletes’ village, to increase accessibility and encourage more participation. This could also address the limitation of having limited time for participation during lunch and dinner hours. Athlete 1 pointed out, “Because athletes are sometimes in a rush to get to their next game, they have to stop by for lunch, eat very quickly, and then go.” Other participants emphasised the importance of promoting the VOA to ensure athletes are aware of it and can participate when available during the Games.

Participants also suggested expanding the program to different countries and occasions beyond the Pacific or Mini Pacific Games, targeting larger populations, which might indicate that they view the VOA as a good practice. Athlete 11 commented, “Do many promotions for the athletes, and then spread the word around the world.” Participants also expressed interest in becoming ambassadors to share the lessons learned with people in their countries. Athlete 77 mentioned that having pamphlets containing key information about each station and core messages to deliver to other athletes and children would be a valuable resource.

## 4 Discussion

The findings of this study offer several implications for athlete support programs while also emphasizing the importance of understanding athletes’ diverse needs and contexts within the Pacific Islands region. Although many participants were unfamiliar with the VOA, the majority expressed interest in learning more about it, suggesting a potential need for increased awareness and promotion among athletes and stakeholders. Support programs such as the VOA should not assume a one-size-fits-all approach; instead, it is crucial for sport managers to critically assess their effectiveness, taking into account the unique contexts and perspectives of athletes at both the exosystem and macrosystem levels (31).

At the exosystem level, the role of the ONOC as a sport governing body in the Pacific Islands region is crucial for the development and implementation of athlete support programs, such as the VOA. Given the pressing need to address the prevalence of non-communicable diseases (NCDs; 54) and human immunodeficiency virus [HIV (55);] in the region, as well as the imperative for athletes to be informed about doping issues and their consequences (56), it is crucial for ONOC to tackle these challenges through targeted educational initiatives. In this context, the VOA serves as a valuable platform for athletes to acquire knowledge about these critical health and ethical issues, while simultaneously empowering them to assume leadership roles within their communities and contribute to the promotion of a healthier, fairer sporting environment. At the macrosystem level, the social and cultural context of the Pacific Islands region plays a significant role in shaping athletes’ experiences and outcomes. For instance, the prevalence of hierarchical relationships with coaches might adversely impact athletes’ safeguarding and wellbeing (57). Researchers have investigated athlete development and performance in relation to various contextual aspects of sport, including exosystem and macrosystem (58, 59). Findings from previous studies highlight the importance of further considering such contextual aspects when supporting athletes, in order to address existing issues and enhance current support systems and programs. These findings are supported by the results of the present study. While the literature emphasizes athlete-centered and athlete-friendly approaches, the present study also highlights the critical role of sport governing bodies in enabling the implementation of athlete-centered approaches through their support initiatives.

By providing education on athletes’ rights, promoting positive coach-athlete relationships, and emphasizing the importance of a nurturing environment, the VOA can contribute to fostering a more supportive and inclusive atmosphere within the realm of high-performance sports in the region. This, in turn, can empower athletes to reach their full potential while ensuring their physical and emotional wellbeing is prioritized. Furthermore, at the macrosystem level, the social and cultural context of the Pacific Islands region influences athletes’ attitudes and behaviors related to environmental responsibility. The recently added *Go Green* station within the VOA attracted participants interested in environmental conservation and eco-friendly lifestyles, who praised the interactive and enjoyable activities that effectively conveyed important messages. The *Go Green* initiative combines educational entertainment with environmental awareness, encouraging athletes to promote sustainability in their communities. This can lead to a wider cultural movement towards environmental care in the Pacific Islands, a region facing urgent challenges such as climate change, pollution, and habitat loss (60). Several studies have focused on educating student-athletes about environmental sustainability and raising their awareness of environmental issues (61–63). These findings highlight the significance of collaborative efforts in providing educational opportunities for student-athletes to engage with environmental issues and consider environmental sustainability, demonstrating a proactive and innovative approach. However, these studies mainly focus on student-athletes attending higher education institutions. In this respect, the present study provides empirical evidence that a sport governing body's support initiative can also serve as a critical channel for educating athletes, regardless of their educational levels.

The identified positive aspects of the VOA, such as learning opportunities, socializing, and increased awareness of athletes’ welfare and wellbeing, highlight the value of such programs in fostering holistic development (3, 64). However, the low awareness of the VOA among participants indicates a need for improved communication and promotion strategies (1). Furthermore, the identified areas for improvement, including language barriers, program accessibility, and the incorporation of more engaging activities, suggest that the VOA and similar programs should be designed with greater consideration for cultural, linguistic, and logistical factors to maximize their reach and impact [Albion and Fogarty, 2003; (15, 65)]. The potential for expanding the scope of the VOA and other athlete support initiatives to engage and educate a broader audience highlights opportunities for growth and innovation. In critically evaluating these findings, it is significant for researchers and sport governing bodies to consider the unique context of sports mega events, as well as the diverse needs and preferences of athletes from different backgrounds (4, 5), to optimize the design and implementation of athlete support programs. Thus, this study serves as a valuable addition to the literature on athlete support, emphasizing the importance of evaluating and adapting such programs based on athletes’ experiences and feedback within the social and cultural context of the Pacific Islands region. This addresses a significant gap identified in the literature, the lack of empirical evidence on assessing exiting athlete support programs (1). Future research should explore a wider range of support services and consider individual, cultural, and contextual factors that influence athletes’ experiences and outcomes, contributing to the holistic wellbeing and long-term success of athletes worldwide.

## 5 Conclusions

The findings add valuable insights to the literature on athlete education, development, and career transition, particularly in under-researched regions. The present study enhances understanding of the field of athlete support programs by addressing critical gaps in the literature. Previous studies [e.g., (1, 19)] have provided limited details about the content and experiences of athlete support programs, especially utilizing a mix-methods approach. By providing a detailed assessment of a program via target population's perceptions, this study offers a comprehensive foundation for investigating similar initiatives. The findings contribute empirical evidence to the literature on career development and transitions in sport, highlighting the fact that athlete support programs should be evaluated to improve service provision and better support athletes’ career development and transitions. The findings also emphasize the value of obtaining participant feedback to refine and advance programs for greater athlete benefit, which address the existing gap in program evaluation research (1). In addition, the findings emphasized the crucial role of sport governing bodies in implementing athlete-centered initiatives and programs, providing clear evidence to the body of knowledge in athlete support programs. Furthermore, in the context of the Pacific Islands, educational levels vary and opportunities for education may be very limited in some nations. Thus, it is worth noting that VOA provides educational opportunities accessible to athletes with all educational levels, which is a significant contribution to the literature.

The quantitative assessment of athletes’ perceptions and attitudes towards the VOA has yielded empirical evidence of potential areas for improvement, which can contribute to its sustainable delivery in the future. The statistical analysis performed (i.e., Pearson Chi-square test and Fisher's exact test) firmly supports the postulation that familiarity with the VOA program is significantly associated with the wiliness to engage further in learning the program. This association suggests that efforts to increase awareness and understanding of the VOA program could be an effective strategy for boosting engagement among the target audience—athletes—in our context. For example, by implementing educational initiatives such as workshops and informational brochures, athletes can gain a deeper understanding of the VOA program's objective and activities. Another example is using strategic collaborative networks such as educational institutions, sports clubs, and community-based organizations. This can lead to a broader demographic of athletes’ awareness of the program, and the networks are important in creating a supportive ecosystem that not only spreads awareness but also integrates the VOA program into the existing networks of these organizations. Future research is recommended to explore the specific aspects of the program that are most appealing to those who are already familiar with it, as well as strategies to increase familiarity among those who are not.

The findings from qualitative research highlight its pedagogic, educational, and informative aspects, which help athletes become more aware of contemporary social issues, empowering them to serve as role models and leaders in their communities. The VOA also has potential for expansion beyond athletes, reaching broader populations. As exposure to the VOA is currently limited to specific occasions such as the Pacific Games, the ONOC, local authorities, and International Olympic Committees (IOC) should consider promoting the VOA to a wider audience and adapting it as a framework for an athlete support program tailored to athletes in other cultural contexts such as Africa, Asia, and South America. Developing written guidelines is also recommended, as they can ensure consistent delivery of content, provide a clear framework for training facilitators, facilitate evaluation and improvement, promote transparency and accountability, and aid in sharing best practices. These guidelines are critical for improving the VOA's effectiveness, ability to scale, and overall impact as it reaches new regions and audiences. This will significantly contribute to its long-term goal of promoting athletes’ wellbeing and leadership on a global scale. The VOA's pedagogic approach, which combines learning with fun and interaction, has proven highly effective in engaging young people. This study contributes to the literature by offering athletes’ first-hand experiences of the VOA, analyzing two different data sets from surveys and interviews.

However, the study has some limitations. One of the limitations is the operationalization of familiarity and interest with the VOA program. For example, familiarity was measured using a categorical variable, which limited responses to a binary choice of “Yes” or “No.” This approach may not capture the nuanced understanding of familiarity. Employing a more psychometrically sound scale (i.e., the use of a developed scale with multiple items) that is designed to measure the variable of familiarity could have been considered. Such a scale would allow for a more refined assessment that could account for varying degrees of familiarity, thus providing more reliable results. Thus, future research is recommended considering the development and validation of a multi-item scale to measure familiarity and interest in the program. Ideally, this scale will be designed to capture a spectrum of familiarity and interest in VOA. Employing such a scale could improve the precision of the measurement and reveal more detailed associations between familiarity and interest, enabling other data analysis. Additionally, longitudinal studies could be employed to assess how familiarity evolves over time and whether it predicts sustained interest and involvement in the VOA program.

As mentioned previously, only athletes from French speaking countries who could speak English were able to participate since the research tools were only available in English, potentially biasing the sample towards English-educated athletes. Thus, this limited the generalizability of the findings. Future research should address language barriers and consider collecting data from smaller island nations to explore diverse cultural and economic contexts. The present study was conducted during the Pacific Games, with the VOA mainly evaluated within the specific context of this event. The limited time available for athletes to engage with the program during breaks between competitions may have influenced their ability to fully experience the VOA. Further investigation of VOA implementation within each country and the effectiveness of locally developed role models can provide valuable insights. Future research can also examine athletes’ in-depth accounts to better understand the VOA and enhance its quality. In this regard, there was no detailed analysis of how the cultural context of different Pacific Island nations might influence the implementation and reception of the VOA. Thus, future studies could examine how these cultural factors impact athletes’ experiences and perceptions of the VOA. Longitudinal studies that examine the long-term outcomes of participation in the VOA could provide insights into whether such involvement leads to sustained changes in behavior, leadership development, or community engagement. Future research applying longitudinal designs would help explore these potential long-term impacts, providing a better understanding of the VOA's value in supporting athletes’ career transitions, mental wellbeing, and life satisfaction.

## Data Availability

The original contributions presented in the study are included in the article/Supplementary Material, further inquiries can be directed to the corresponding author.
